# Simple dynamic cell culture system reduces recording noise in microelectrode array recordings

**DOI:** 10.1557/s43579-024-00554-3

**Published:** 2024-04-22

**Authors:** Darius Hoven, Misaki Inaoka, Reece McCoy, Aimee Withers, Róisín M. Owens, George G. Malliaras

**Affiliations:** 1 Electrical Engineering Division, Department of Engineering, University of Cambridge, Cambridge, CB3 0FA UK; 2 Department of Chemical Engineering and Biotechnology, University of Cambridge, Cambridge, CB3 0AS UK

**Keywords:** Bioelectronic, Biomedical, Devices, Fluidics, Microelectronics, Microscale

## Abstract

Microelectrode arrays (MEAs) have applications in drug discovery, toxicology, and basic research. They measure the electrophysiological response of tissue cultures to quantify changes upon exposure to biochemical stimuli. Unfortunately, manual addition of chemicals introduces significant noise in the recordings. Here, we report a simple-to-fabricate fluidic system that addresses this issue. We show that cell cultures can be successfully established in the fluidic compartment under continuous flow conditions and that the addition of chemicals introduces minimal noise in the recordings. This dynamic cell culture system represents an improvement over traditional tissue culture wells used in MEAs, facilitating electrophysiology measurements.

**Graphical abstract:**

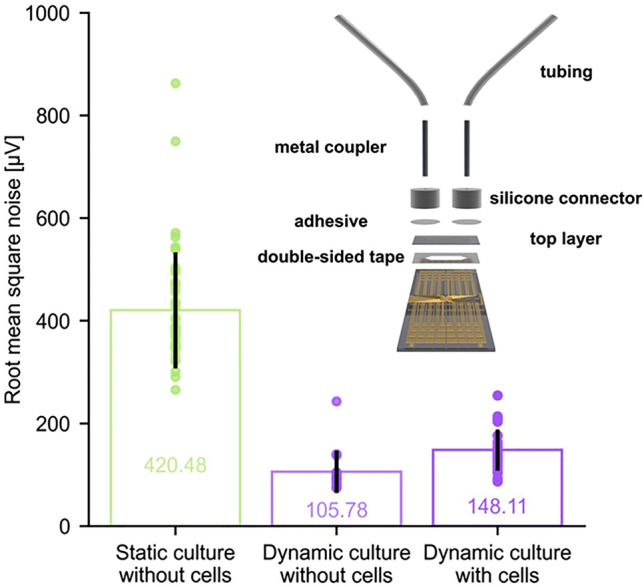

## Introduction

Microelectrode arrays (MEAs) are powerful tools in clinical practise and research as they facilitate parallelised electrophysiology recordings from diverse cell types and offer insights into cellular processes and communication within the human body.^[1,2]^ Recordings can be conducted either *in vivo*, by placing MEAs directly into the living organism, or *in vitro*, by culturing tissues on top of the MEA. The latter is of particular interest due to its reduced complexity in comparison to the native organism, allowing for controlled cell physiology investigations addressing various research questions, especially in the field of neuroscience.^[3]^ MEAs also have significant applications in drug discovery and toxicology, especially in conjunction with human-derived tissue cultures.^[4,5]^

The primary scientific challenge in *in vitro* tissue cultures lies in the faithful replication of relevant characteristics inherent to *in vivo* systems, striving for a balance between simplicity and accuracy to ensure cost- and time-efficiency, thereby enhancing biological relevance and statistical robustness of experimental outcomes.^[6]^ Usually, tissue cultures on MEAs are established in a well that is placed on top of the electrodes.^[7]^ Candidate drugs, potentially harmful chemicals and other stimuli, are pipetted into the well to observe their effects on the electrophysiological response of the cells. While this approach is simple and akin to traditional cell culture in T-flasks and well plates, it lacks delicate and precise control of the cell microenvironment and requires changing large media volumes using pipettes. Due to poor mixing in static volumes, large concentration gradients, and long diffusion distances, pipetting introduces significant transient effects. Together with the electrical disturbances caused by the approach and retraction of the pipette, they lead to significant noise being induced in the electrophysiology recordings.

An efficient and precise way to control the environment around cells is enabled by microfluidics.^[6,8]^ Microfluidics allows the manipulation of small liquid volumes, opening new possibilities and entire fields of research like lab- or organ-on-a-chip due to small sample volumes and fine control over fluidic components.^[9,10]^ This fine control applied in microfluidic cell cultures has been shown to facilitate relevant *in vitro* models by providing physiological relevant shear stress to specific cell lines and allowing for multi-parametric in-line cell monitoring.^[11–14]^ Additionally, microfluidic systems offer powerful means for bottom-up engineering of neuronal cells and circuits, and recent research has focused on integrating microfluidics with 3D cell culturing techniques to more accurately replicate the complexities of the native cell environment.^[15,16]^ However, this enhanced control comes at the expense of an increased complexity in fabrication and operation of such systems, which impedes their widespread adoption as a laboratory standard.^[6]^ Besides, careful consideration of the system’s chemical and physical characteristics becomes paramount in microfluidic channels. Materials selection directly influences unintended component exchange between media and channel and mitigates the risk of bubble formation, while appropriate coordination of channel dimensions and flow rates ensures shear stress to stay at physiologically relevant levels.^[16,17]^

In this paper we introduce a facile method to establish and modify cell cultures on microelectrode arrays using fluidic channels. We fabricate the fluidic system in a highly customisable fashion using laser-cut double-sided tape, and actuate it using a standard syringe pump. We show that cells can successfully be cultured under continuous flow in the dynamic system. We further show that our approach mitigates electrical interference in electrophysiology recordings arising from the addition of chemical stimuli. This means that the proposed dynamic cell culture system provides a facile and viable alternative to static cell cultures on microelectrode arrays.

## Materials and methods

### Static and dynamic cell culture systems

**Figure 1:**
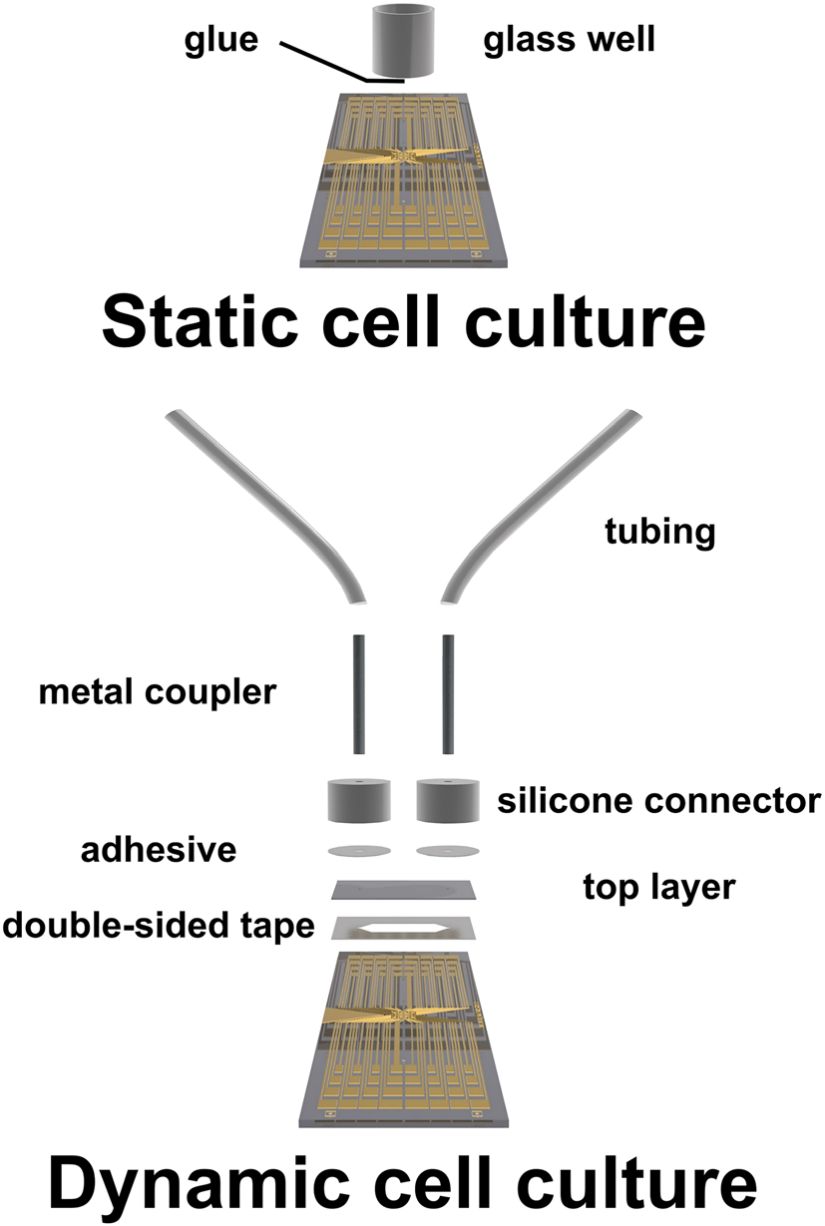
Static and dynamic cell culture system on microelectrode arrays. Static cell culture can be implemented by confining cells in a glass well placed on top of the electrodes. Cell media must be manually exchanged periodically and application of biochemical stimuli for experimental purposes is performed by pipetting. In contrast, dynamic cell culture is enabled by microfluidics. Microfluidic channels can be fabricated on microelectrode arrays by stacking laser-cut double-sided tape and a polymeric top layer. Connectors and couplers allow for connecting the microfluidic channel via tubes to a syringe pump for continuous media perfusion. This also allows to precisely change the cells’ microenvironment during experiments.

Schematics of the static and dynamic cell culture systems are shown in Fig. 1. 64 channel MEAs featuring 62 circular microelectrodes (100 μm diameter) were fabricated using a lithographic process in accordance with a previously reported protocol.^[18]^ The Au electrodes were coated with poly(3,4-ethylenedioxythiophene) doped with polystyrene sulfonate (PEDOT:PSS) to reduce impedance.^[19,20]^ The MEAs were oxygen plasma treated, cleaned with 70 v/v% ethanol, and blow dried prior to attachment of the cell culture system.

For the static cell culture system, a well was created on the MEA by attaching a cylindrical glass (9 mm inner diameter, 10 mm height) with polydimethylsiloxane glue (PDMS, Sylgard 184, Dow).

For the dynamic cell culture system, 160 μm double-sided medical tape (3 M 1522, 3 M) and a 175 μm poly(methyl methacrylate) sheet (Goodfellow Cambridge Ltd) were laser cut (Universal Laser Systems) according to the desired channel layout (area ∼ 74.8 mm^2^) and stacked to define the microfluidic channel. Silicone connectors (Press Fit Tubing Connectors, Grace Bio-Labs) and metal couplers (metal part of Blunt-end Luer Lock Syringe Needles, Darwin Microfluidics) were used to connect the channel outlet via silicone tubing (Tygon ND 100-80 tubing, Compagnie de Saint-Gobain) with a 3 ml plastic syringe (BD Plastipak 3 ml Syringe, Beckton Dickinson), which is actuated using a syringe pump (Pump 33 DDS Syringe Pump, Havard Apparatus). The inlet was connected via a commercial silicone connector and polytetrafluoroethylene tubing (PTFE Tubing 1/32″ OD × 0.30 mm ID for Microfluidics, Darwin Microfluidics) to a 4 ml glass vial (Ossila Ltd) holding cell media. Holes were drilled into the vial cap to attach a 0.22 μm syringe filter and tubing fixed with glue to complete the setup.

### Cell culture

We used human 1321N1 astrocytoma cells as a model cell line. Astrocytes are currently attracting a great deal of interest, as recent evidence suggest that they are involved in information processing in the brain.^[18]^ Importantly, astrocytes generate much weaker electrical activity than neurons, which necessitates a highly sensitive electrophysiology system to detect. They are, thus, a better model system for testing noise reduction in electrophysiological recordings.

Human 1321N1 astrocytoma cells were purchased from the European Collection of Authenticated Cell Cultures and grown in flasks with high glucose Dulbecco’s modified eagle medium (Sigma-Aldrich) supplemented with 10 v/v% foetal bovine serum (Life Technologies-Invitrogen), 1 v/v% Glutamax (Life Technologies), and 1 v/v% penicillin/streptomycin (Invitrogen).

The cell culture systems were sterilised for 10 min with 70 v/v% ethanol followed by a soaking step in phosphate buffered saline (Dulbecco’s phosphate buffered saline, Sigma-Aldrich), and media for 20 min each. Cells were seeded at 30,000 cells/cm^2^ and cultured in a humidified incubator at 37°C and 5% CO_2_ for 2 days prior to experimentation. Media was continuously exchanged in the dynamic system, with a flow rate of 0.25 μl/min matching two to 3 days media renewal intervals under static conditions but minimising the applied shear stress on cells. In the static system, media was renewed after 2 days using a pipette.

### Calcium imaging

Intracellular calcium imaging was performed by incubating cells with 10 μM calcium indicator (Calbryte 520 AM, AAT Bioquest Inc.) for 1 h, followed by carefully washing with phosphate buffered saline, and replacing the buffer with fresh media. Fluorescence images were recorded with a confocal microscope (LMS 800, Carl Zeiss AG) in the absence and presence of 80 µM 4-Aminopyridine (4-AP).

### Impedance measurement

Impedance magnitude and phase at 1 kHz were measured with an electrophysiology amplifier (128 channel RHS stimulation/recording system, Intan Technologies). We defined electrodes as operational if impedance magnitudes at 1 kHz fall within the range of 1 to 100 kΩ. Moreover, recordings from one of two ports from the dynamic cell culture condition without cells had to be removed due to a connection issue. Operational electrodes for the static system without cells showed 17.25 ± 9.04 kΩ impedance magnitude and − 32.34 ± 5.99° phase at 1 kHz compared to 5.66 ± 1.39 kΩ and − 24.39 ± 5.5° for the dynamic system. As expected, the presence of cells introduces impediments along the path to the electrodes resulting in increased impedances seen in the dynamic system with cells of 16.47 ± 9.44 kΩ and − 20.16 ± 4.77°.

### Electrophysiology recordings

Recordings were performed with an electrophysiology amplifier (128 channel RHS stimulation/recording system, Intan Technologies). The setup was placed in a Faraday room to reduce environmental noise. Signals were recorded at 20 kHz sampling frequency with analogue cut-offs at 0.1 and 7500 Hz and notch filtered at 50 Hz. 80 µM 4-AP was added to chemically stimulate cells. Additional control recordings were performed without cells, with the culture systems filled with media.

### Data and statistical analysis

Fiji was used to calculate mean grey values of the captured fluorescence images taken with sampling frequency of 1 Hz for 3 individual cells and fluorescence intensities were normalised to the first image. Durations of intensity spikes were detected using Python by calculating the 95% spike width for spikes above a normalised intensity of 1.1. Electrophysiology data was processed using manufacturer provided software packages (Intan Technologies) and detrended as well as filtered with a 50 Hz notch and 100 Hz low-pass filter before further analysis. Additionally, data were downsampled from 20 kHz to 200 Hz to reduce storage space and processing time. Electrical noise was defined as voltage peaks exceeding ± 50 μV and quantified for each recording electrode as root mean square of all detected spike amplitudes.

Additional data analysis was performed in Python and statistical tests (Kruskal–Wallis test, post hoc Dunn’s test; *α* = 0.05, significance for *p* ≤ 0.05) for non-normally distributed data (Shapiro–Wilk normality test) in R. Results are given as mean ± standard deviation. Figures were created using a combination of Python, Inkscape, and Shapr3D.

## Results

**Figure 2:**
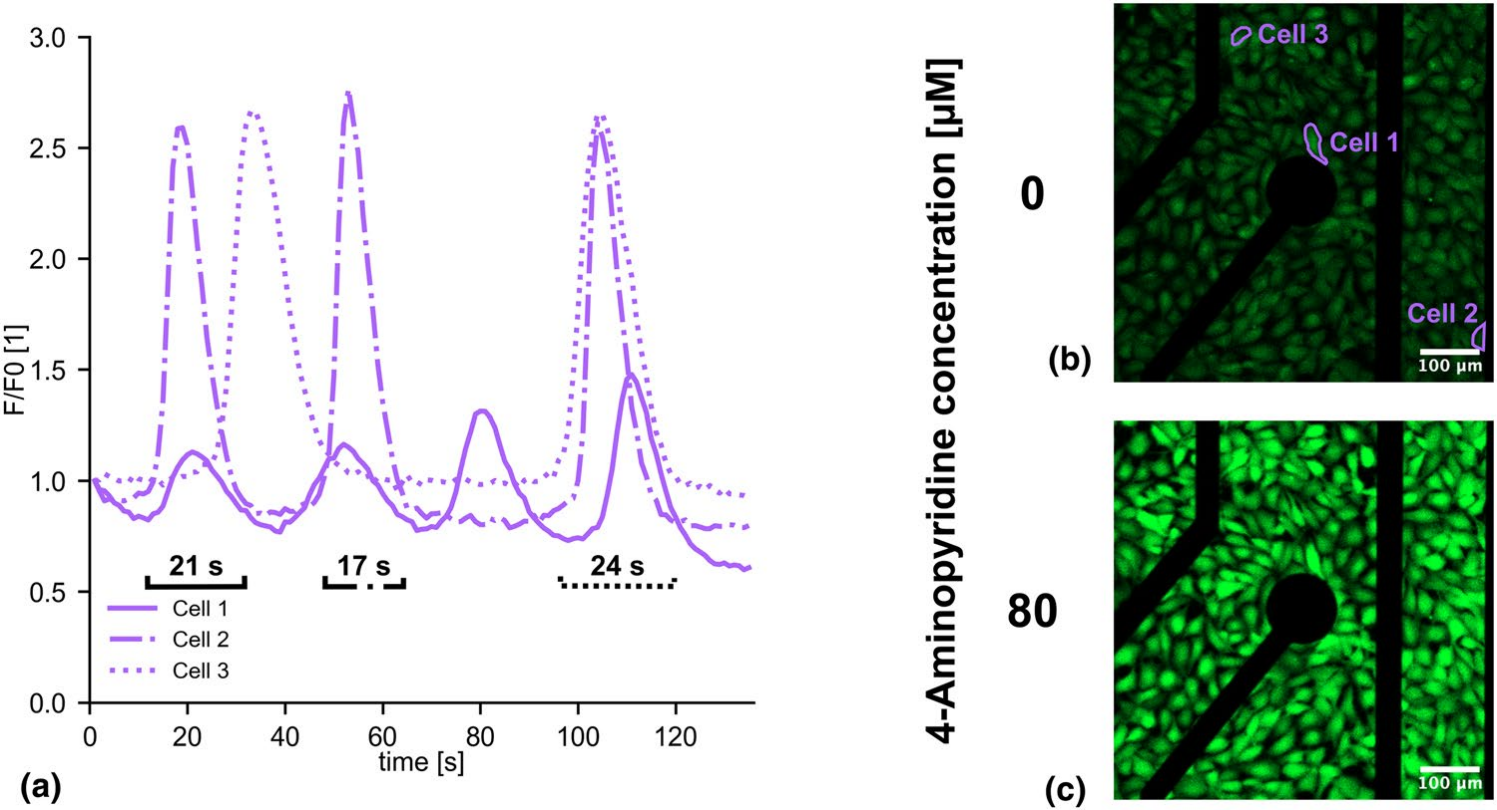
Viability assessment of 1321N1 human astrocytoma cells cultured on microelectrode arrays under dynamic conditions. (a) Spontaneous variation of normalised fluorescence intensity over time indicates normal activity of unstimulated cells [Cells 1–3 labelled in (b)] in microfluidic channel featuring 21.59 ± 3.96 s spike durations. (b, c) Fluorescence images visualise intracellular calcium in cells stained by Calbryte 520 AM calcium dye either (b) unstimulated or (c) chemically stimulated with 80 µM 4-Aminopyridine. Images were taken 2 days after seeding.

We performed calcium imaging to verify function. Figure 2(a) shows the spontaneous intracellular calcium activity of 3 1321N1 astrocytoma cells in dynamic culture. The outline of the cells is indicated in Fig. 2(b). The normalised fluorescence intensity shows periods of increased intracellular calcium concentration persisting for an average duration of 21.59 ± 3.96 s, corresponding to a frequency around 0.05 Hz. This finding aligns with existing literature on spontaneous astrocyte activity.^[21,22]^ We further exposed the astrocytes to 80 µM 4-Aminopyridine (4-AP), a chemical known to stimulate astrocytes and enhance calcium wave activity.^[21,23]^ As expected, this led to a higher fluorescence intensity [Fig. 2(c)]. The dynamic culture system is therefore capable of establishing and sustaining a cell culture, and is compatible with traditional imaging methods used in biology.

**Figure 3:**
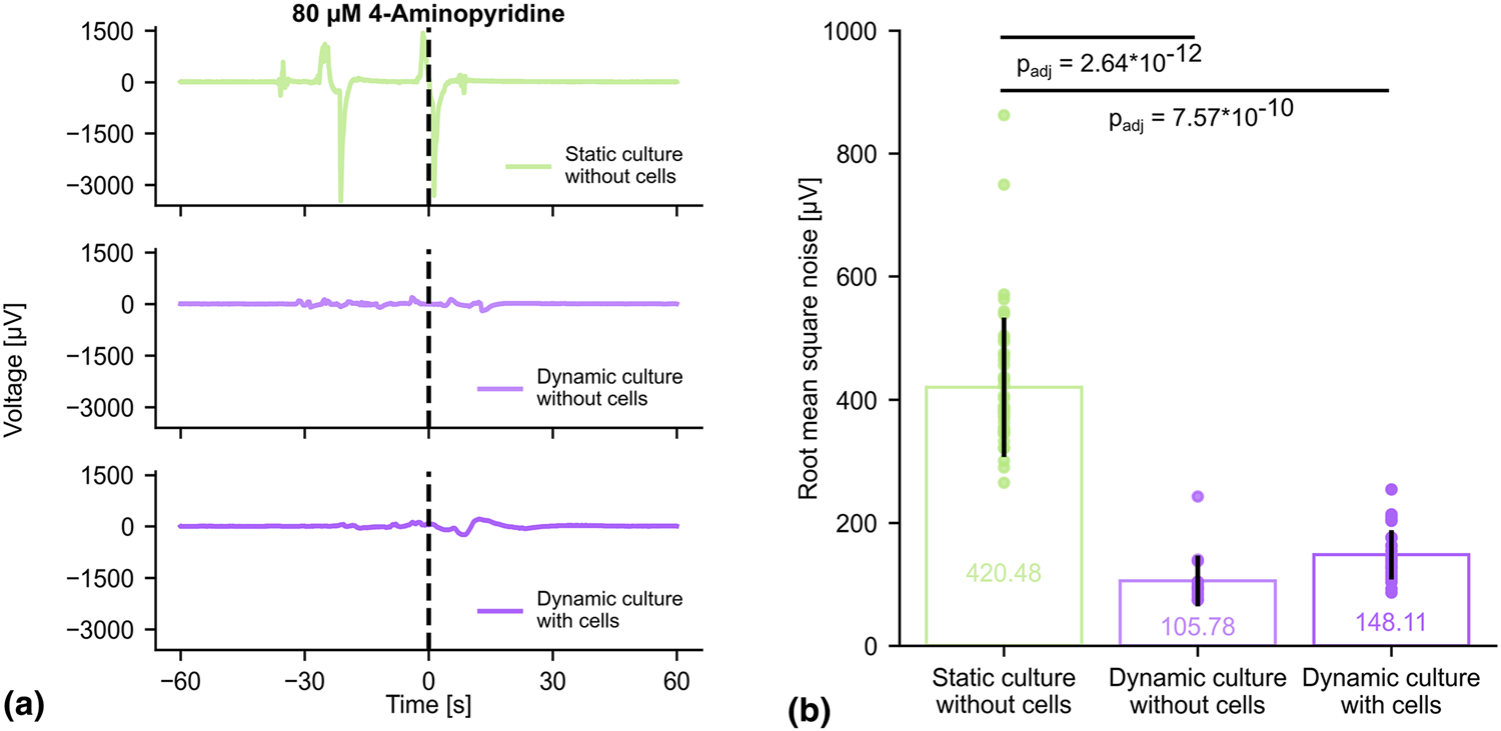
Electrophysiology recordings in static and dynamic cell culture system. (a) Representative signal traces are shown for electrophysiology recordings in static (light green first row) and dynamic (light violet second row) cell culture systems without cells as well as dynamic (violet third row) cell culture system with cells when injecting 80 µM 4-Aminopyridine at 0 s. Signals were filtered (50 Hz notch filter, 100 Hz low-pass filter, downsampled from 20 kHz to 200 Hz) and detrended for further analysis. (b) Electrical noise is calculated as root mean square of recorded voltage spikes exceeding ± 50 μV and summarised (mean ± standard deviation) for all functioning circular electrodes (opening diameter 100 μm, static cell culture system: *n* = 44, dynamic cell culture system: without cells *n* = 16, with cells *n* = 28). While no significant difference can be observed when comparing the dynamic culture without and with cells (*p*_adj_ = 6.76 × 10^–2^), the static system shows significantly higher noise (Kruskal–Wallis test, post hoc Dunn’s test) compared to the dynamic system without cells (*p*_adj_ = 2.64 × 10^–12^) or with cells cultured for 2 days prior to experimentation (*p*_adj_ = 7.57 × 10^–10^).

We subsequently performed electrophysiology recordings to assess the ability of microfluidics in reducing electrical noise arising from chemical stimulation during experiments. Astrocytes show electrophysiological activity in form of hyperpolarised membranes and calcium signalling, which can be stimulated by 4-AP. As such, we pipetted 80 µM 4-AP during recordings in a static cell culture system filled with media. Figure 3(a) shows a representative recording, revealing a dramatic increase in noise as the pipette approaches the well and dispenses the solution. The peak noise is shown to exceed 3 mV. In sharp contrast, the noise elicited in the dynamic culture system is considerably smaller. As discussed below, this noise corresponds to a person approaching the setup to operate the syringe. The noise increases somewhat when cells are present, consistent with the higher value of electrode impedance, but the effect is rather minimal and potentially a consequence of deviation in syringe operation. The root mean square values calculated by accounting for all voltage spikes exceeding ± 50 μV, are shown in Fig. 3(b). Statistical analysis (Kruskal–Wallis test, post hoc Dunn’s test) showed a significant reduction in noise for recordings performed in the microfluidic channel without and with cells (without cells: 105.78 ± 41.21 μV, *p*_adj_ = 2.64 × 10^–12^; with cells: 148.11 ± 40.06 µV, *p*_adj_ = 7.57 × 10^–10^) compared to the static well (420.48 ± 113.10 μV), but no significant difference when comparing the dynamic system without and with cells (*p*_adj_ = 6.76 × 10^–2^).

These results demonstrate the viability of the dynamic system to establish cell cultures under continuous media perfusion and significantly reduce electrical noise arising from addition of chemical stimulants during electrophysiology recordings.

## Discussion

Tissue culture in a well attached to the MEA represents the most common configuration for *in vitro* electrophysiology measurements. Despite its convenience, it leads to electrical noise in recordings during the addition of chemical stimulants. Here, we introduced a simple, lithography-free, dynamic culture method characterised by continuous media exchange. By using a syringe pump to actuate the liquid exchange and fabricating the microfluidic channel by laser cutting double-sided tape and polymeric top layers, we presented a rapid and highly flexible prototyping approach capable of creating various designs tailored to bespoke MEA designs. For larger features, the laser cutter can be replaced with a cost-efficient blade cutter, making all materials and tools readily available to most laboratories.

The dynamic culture approach offers significant advantages. Continuous media exchange actuated by a syringe pump removes the need for manual media exchange, improving cell culture efficiency, maintaining near-constant media composition, and reducing the risk of contamination. Microchannels offer a better control over the cells’ microenvironment and provide an improved, user-friendly handling experience when applying chemical stimulation. This prevents having to move the MEA from an imaging setup in order to pipette stimulants, and micromotions associated with the manual addition of fluid in the well.

Our findings demonstrate the viability of culturing 1321N1 astrocytoma cells under dynamic culture conditions. By employing calcium imaging, we observed the calcium wave activity from cells cultured in our system to be comparable to that of cells cultured under standard static culture conditions. Subsequent electrophysiology recordings demonstrated that the dynamic culture system shows a significant reduction in electrical noise levels during the addition of a chemical stimulant. This is especially relevant under conditions where the electrophysiology activity is very weak.^[1]^ It is important to note that we did not observe discernible electrical activity from astrocytes. This is because the electrophysiology amplifier cuts off frequencies below 0.1 Hz, therefore cannot pick up calcium waves, which correspond to the main electrical activity of astrocytes.^[22]^ The absence of innate activity made it easier to quantify the noise, validating our choice of astrocytes as a good model for this investigation.

While our work offers a compelling proof of concept, highlighting the advantages of dynamic cell culture, there is room for improvement in several aspects. We perceived electrical noise already before as well as after injecting the chemical stimulant, which can be seen in Fig. 3(a) within the intervals of − 30 to 0 s and 0 to 15 s, respectively. It is crucial to avoid or at least significantly reduce noise levels even further to prevent potential negative interferences resulting in distortion or masking of the signals of interest. We assign this noise primarily to the experimental design requiring entry into the Faraday room to apply the chemical stimulant and, in case of the static system featuring a culture well, the removal of liquid prior to the addition of fluid holding the chemical stimulant to reach a homogenous distribution of the desired concentration in the well. The presence of an individual approaching, interacting with, and withdrawing from the system appears to cause an artefact that results into a considerable source of noise during sensitive recordings, which might even explain the remaining noise arising from chemical injection. While using longer tubing or a remotely controlled syringe pump to administer stimuli from outside the recording room might be an easy prevention of this noise source for the dynamic cell culture system, replacing the manual pipetting for the static system is more difficult.

Moreover, we occasionally detected bubble formation and subsequent cell detachment at the air–liquid interface when culturing cells over extended periods in the microfluidic channel. Although this was not observed during the electrophysiological recordings, it still impacts the system’s reliability by risking contamination or interfering with cell distribution on the MEA, which is especially relevant for long time experiments investigating network effects. Thus, future endeavours should focus on achieving a more effective sealing of the microfluidic setup through correct material selection, effective engineering, and secure channel-tube connections to prevent bubble formation and cell detachment. We anticipate improving the signal-to-noise ratio by a synergy of such refinements with the selection of appropriate recording hardware and experimental design, thereby enabling successful recordings and fostering a deeper understanding of the crucial role played by astrocytes in information processing within the brain.^[18,21]^

In summary, we would like to emphasise that like most techniques, dynamic cell culture may not offer an optimal solution for every cell line and experimental research question. The improved control over experimental conditions, specifically the ability to precisely modulate the cells’ microenvironment through microfluidics, comes at the expense of increased complexity in setup fabrication and operation—a complexity we have sought to significantly reduce here. We envision our work to provide a viable alternative to standard cell culture in a well on microelectrode arrays because of its simplicity relative to other microfabrication techniques, accessibility, and improved experimental control.

## Data Availability

All data for the work are included in this manuscript and datasets generated and/or analysed during the current study are available from the corresponding author upon reasonable request.
